# Segment hepatic herniation through a diaphragm defect simulating basal lung nodes

**DOI:** 10.1186/1749-8090-8-S1-P150

**Published:** 2013-09-11

**Authors:** F Gradica, A Menzelxhiu, A Cami, F Kokici, I Peposhi, E Fype, I Skenduli, L Lisha, A Leka, D Argjiri

**Affiliations:** 1 Thoracic Surgery Department, University Hosptal "Shefqet Ndroqi", Tirana, Albania; 2 Anesthesiology and Reanimation Department, University Hosptal "Shefqet Ndroqi", Tirana, Albania; 3 Pneumology Department, University Hosptal "Shefqet Ndroqi", Tirana, Albania; 4 Imagery Department, University Hosptal "Shefqet Ndroqi", Tirana, Albania

## Background

A resection procedure with a diagnostic goal is indicated depending on the clinical and epidemiological features of the lung nodes of the patient. Our objective was to introduce a patient diagnosed with left lobe liver abscess where extensive study showed a lung node not visualized previously.

## Methods

A 51 year old female non-smoker, who had high blood pressure, was diagnosed with left lobe liver abscess, was suspicioned as carcinoma of pancreatic head. Definitive diagnosis was liver abscess and basal right lung node. Additional thoracic tests included a CT without contrast showing a node in the right lung inferior lobe, measuring 4x3 cm in diameter anterio-posteriorly and 4x5 cm in diameter transversally. Respiratory function tests were optimal; thus it was decided to achieve a diagnostic-therapeutical surgical procedure of the lung nodes in order to make a differential diagnosis of primary pulmonary tumor or metastatic lesions of another type of lesion in liver.

## Results

A right posterolateral thoracotomy was performed. After separating the lung ligament and adhesyolysis diaphragmatic part of parietal pleura, a nodus mass was observed in the diaphragm, alongside a defect in the diaphragm several centimetres wide with liver tissue prolapse that corresponded to the preoperative CT. The liver segment was pushed back into the abdomen and the diaphragm defect was sewn-over and the thorax closed. The patient did not have any history of trauma that might justify the diaphragm injury; thus it was assumed that this was a congenital defect not previously diagnosed.

## Conclusions

It is not always possible to diagnose a lung node through non –surgical procedures because CT scan can mimic diaphragm defects and incisional hernias of intra abdominal organs as lung nodes. Thoracoscopy, as initial step in surgical procedures with diagnostic goals, might help prevent unnecessary and more aggressive procedures.

